# Immunological Approaches Towards Cancer and Inflammation: A Cross Talk

**DOI:** 10.3389/fimmu.2018.00563

**Published:** 2018-03-20

**Authors:** Xinglong Qu, Ying Tang, Shucheng Hua

**Affiliations:** ^1^Department of Respiration, The First Hospital of Jilin University, Changchun, China

**Keywords:** immune system, cancer, inflammation, cytokines, tumor-associated neutrophils, tumor-associated macrophages, T cytotoxic cells, T helper cells

## Abstract

The inflammation is the protective response of the body against various harmful stimuli; however, the aberrant and inappropriate activation tends to become harmful. The acute inflammatory response tends to resolved once the offending agent is subside but this acute response becomes chronic in nature when the body is unable to successfully neutralized the noxious stimuli. This chronic inflammatory microenvironment is associated with the release of various pro-inflammatory and oncogenic mediators such as nitric oxide (NO), cytokines [IL-1β, IL-2, interleukin-6 (IL-6), and tumor necrosis factor-α (TNF-α)], growth factor, and chemokines. These mediators make the inflammatory microenvironment more vulnerable toward tumorigenesis. The pro-inflammatory mediators released during the chronic inflammation tends to induce several molecular signaling cascades such as nuclear factor kappa B, MAPKinase, nuclear factor erythroid 2-related factor 2, phosphoinositide-3-kinase, Janus kinases/STAT, Wnt/B-catenin, and cyclic AMP response element binding protein. The immune system and its components have a pleiotropic effect on inflammation and cancer progression. Immune components such as T cells, natural killer cells, macrophages, and neutrophils either inhibit or enhance tumor initiation depending on the type of tumor and immune cells involved. Tumor-associated macrophages and tumor-associated neutrophils are pro-tumorigenic cells highly prevalent in inflammation-mediated tumors. Similarly, presence of T regulatory (Treg) cells in an inflammatory and tumor setting suppresses the immune system, thus paving the way for oncogenesis. However, Treg cells also inhibit autoimmune inflammation. By contrast, cytotoxic T cells and T helper cells confer antitumor immunity and are associated with better prognosis in patients with cancer. Cytotoxic T cells inflict a direct cytotoxic effect on cells expressing oncogenic markers. Currently, several anti-inflammatory and antitumor therapies are under trials in which these immune cells are exploited. Adoptive cell transfer composed of tumor-infiltrating lymphocytes has been tried for the treatment of tumors after their *ex vivo* expansion. Mediators released by cells in a tumorigenic and inflammatory microenvironment cross talk with nearby cells, either promoting or inhibiting inflammation and cancer. Recently, several cytokine-based therapies are either being developed or are under trial to treat such types of manifestations. Monoclonal antibodies directed against TNF-α, VEGF, and IL-6 has shown promising results to ameliorate inflammation and cancer, while direct administration of IL-2 has been shown to cause tumor regression.

## Introduction

Inflammation has become an important hallmark of cancer, and elevated inflammatory mediators are related to poor prognosis in patients with cancer (1). Similarly, an increase in the number of immune cells such as tumor-associated neutrophils (TANs) are related to poor outcomes in patients with glioblastoma, renal cell carcinoma (RCC), melanoma, colorectal cancer, hepatocellular carcinoma (HCC), pancreatic ductal carcinoma, and head and neck cancer (2, 3). Furthermore, inflammation secondary to prolonged infection is also linked to oncogenesis. Several experimental and clinical studies have demonstrated that the majority of deaths that occur from cancer are connected with chronic unresolved infections (4). Similarly, some types of chronic inflammation that are not caused by infectious agents can also initiate tumor development. Pancreatic, esophageal, and gall bladder tumors are promoted by inflammatory conditions, including Barrett’s metaplasia, esophagitis, and chronic pancreatitis, respectively (4).

According to numerous reports, several researchers have attempted to establish a formal relationship between inflammation and cancer since the eighteenth century (5). However, this connection was only formalized by the observation of uncontrolled cell proliferation at the site of inflammation and the existence of inflammatory cells at the tumor site (6). Likewise, inflammatory mediators and reactive nitrogen oxygen species (RNOS) with resultant elevation of the nuclear factor kappa B (NF-κB) pathway and cyclooxygenase-2 (COX-2) activity may facilitate inflammation-mediated tumorigenesis. Prolong inflammation may also trigger altered expression of oncogenes and tumor suppressor genes (5). The occurrence of germline mutations is rare, while their association with somatic and environmental factors is more common (7). Chronic inflammation is the most important environmental factor, posing higher risk of tumor initiation (7).

According to the previous studies, it is established that infiltration of the tumor microenvironment by leukocytes promotes the development and progression of various tumors, including epithelial tumors (8). Conversely, some subsets of leukocytes such as natural killer (NK) cells and cytotoxic T cells (CTLs) have established antitumor activity (9). Certain tumors such as breast cancer have shown increased levels of cells of both the innate and acquired immune systems, such as B cells, T cells, and macrophages (10). Similarly, the level of inflammatory cytokines such as interleukin-6 (IL-6) was increased in carcinoma of the pancreas (11). Furthermore, marked elevation of the level of IL-1β was related to a more advanced and aggressive nature of the disease (12). The inflammatory immune response may be tumorigenic or anti-tumorigenic, depending on the appropriate and balanced activation of both adaptive and innate arms of the immune system (5). A well-regulated acquired immune response is considered to be anti-tumorigenic, whereas inappropriate innate or acquired immune system stimulation can cause chronic inflammation and resultant progression toward oncogenesis (5). The inflammatory response is resolved after downregulation of pro-inflammatory cytokines and by expression of IL-10 anti-inflammatory cytokines (13). An anti-inflammatory role of IL-10 has been demonstrated in mice lacking the IL-10 gene, wherein colonic inflammation spontaneously develops into colon cancer (13).

## Types of Inflammation and Involvement of Immune Cells

Inflammation is a protective mechanism initiated in response to tissue damage caused by infection, trauma, and chemical exposure (4). At the beginning of inflammation, neutrophils are the first cells to infiltrate under the direction of inflammatory mediators released at the site of inflammation (1). As the process of inflammation proceeds, various other players of inflammation such as lymphocytes and macrophages are stimulated and recruited to the inflammatory setting. This immune infiltration is facilitated by the increased expression of chemokines, growth factors, and cytokines released (14). The major aim of these cells is to boost the body’s defense process with the addition of neutralizing the offending agent (14).

However, when the body is unable to resolve the acute inflammatory response, this leads to chronic and persistent inflammation (15). This chronic and persistent inflammation can trigger the development of various types of tumors such as colorectal carcinoma after chronic inflammatory bowel disease (IBD) (15). Similarly, the chronic inflammatory microenvironment set by persistent *Helicobacter pylori* infection induces gastric cancer and mucosa-associated lymphoid tissue cancer. Chronic HBV and HCV virus infections increase the likelihood of HCC development (16–18). Similarly, a connection between colon cancer and bladder cancer in patients with chronic and persistent *Schistosoma* and *Bacteroides* infections has been reported (15). In addition, several environmental factors such as tobacco smoking cause chronic obstructive pulmonary disease increasing the likelihood of lung cancer development (7, 19). Similarly, other environmental factors such as silica or asbestos exposure can also trigger cancer by inducing the synthesis of pro-inflammatory cytokines (20), and even inflammation associated with obesity increases cancer risk by 1.6 times (21). By contrast, administration of non-steroidal anti-inflammatory drugs (NSAIDs) in randomized controlled studies has reduced the incidence of colon cancer in familial adenomatous polyposis patients. Similarly, a notable decline was observed in the incidence of lung cancer in chronic smokers following NSAID usage (22).

The macrophages and neutrophils are competent phagocytic cells and considered first line of defense against the offending agent. Generally, it was believed that neutrophils are the cells of acute inflammation, whereas monocytes were recognized the cells of chronic inflammation. However, several studies reported that involvement of neutrophils in adaptive immune response to resolve the chronic inflammation and also implicated the involvement of monocyte/macrophages in acute inflammatory response (23). The neutrophils following recruitment to the acute inflammatory site are activated, kill and phagocytes the invading agent and associated with the release of inflammatory mediators such as cytokines to recruit monocytes. The recruited monocytes undergo differentiation to macrophages and propose a bimodal transformation of immune cells from neutrophils to monocytes (24, 25). However, several studies suggest that chemoattractant like MCP-1 produced at the inflammatory site by tissue macrophages induces the recruitment of monocytes regardless of the presence of neutrophils at inflammatory site (25). Thus, it can be postulated that neutrophils and monocytes interplay between innate and immune system and trigger several functions such opsonization, release of inflammatory mediators, differentiation of Th1 cells, and the chemotaxis of Th1 and Th17 cells (26). NK cells are considered lymphocytes based on their morphology, their origin from the bone marrow, and the expression of lymphoid-associated molecules. However, NK cells lack antigen-specific cell surface marker and are also considered the cells of innate immune defense system. NK cells are non-specific in nature and works against the virus infection such as infection of herpesvirus (27). CD4^+^ Th1 cells and CD8^+^ T cells associated with the release of INF-γ critically regulate the tumor immunity by killing and impending cancer growth. Furthermore, the lymphocytic infiltration into the tumor microenvironment is related to better prognosis (22, 28). The CD8^+^ T cells also mediate antitumor effect by direct cytotoxicity. However, all T cells are not associated with antitumor immunity because CD4^+^ T cells expressing master transcription factor Foxp3 (CD4^+^CD25^+^Foxp3^+^) and CD25 termed as regulatory T cells (Tregs), promote tumor growth by reducing the immune responses (22). The basic aim of these cells is to inhibit the activation of effector immune cells against the self-antigen, reduce the chances of autoimmune responses, and inhibit inflammatory responses during the normal conditions. The studies shows that Tregs cells is associated with the inhibition of both CD4^+^ and CD8^+^ T cells mediated immunity and this inhibition of CD4^+^ and CD8^+^ T cells within the tumor microenvironment reduces tumor immunity (22). Th17 producing CD4^+^ cells are involved in the severe autoimmune disease and chronic persistent inflammatory conditions. Furthermore, Th17 secreted IL-23 is key factor maintaining and expanding Th17 inflammatory cell count. The IL-23 has been recently reported to have pro-tumorigenic effect, promoting inflammation, angiogenesis, and reducing the CD8^+^ T cell within the cancer microenvironment. Furthermore, the existence of Th17 within the tumor microenvironment is associated with the inhibition of INF-γ producing CD4^+^ cell (22).

## Inflammatory Mediators Mediated Genetic Alteration; Paving the Way for Tumorigenesis

**Figure 1:**
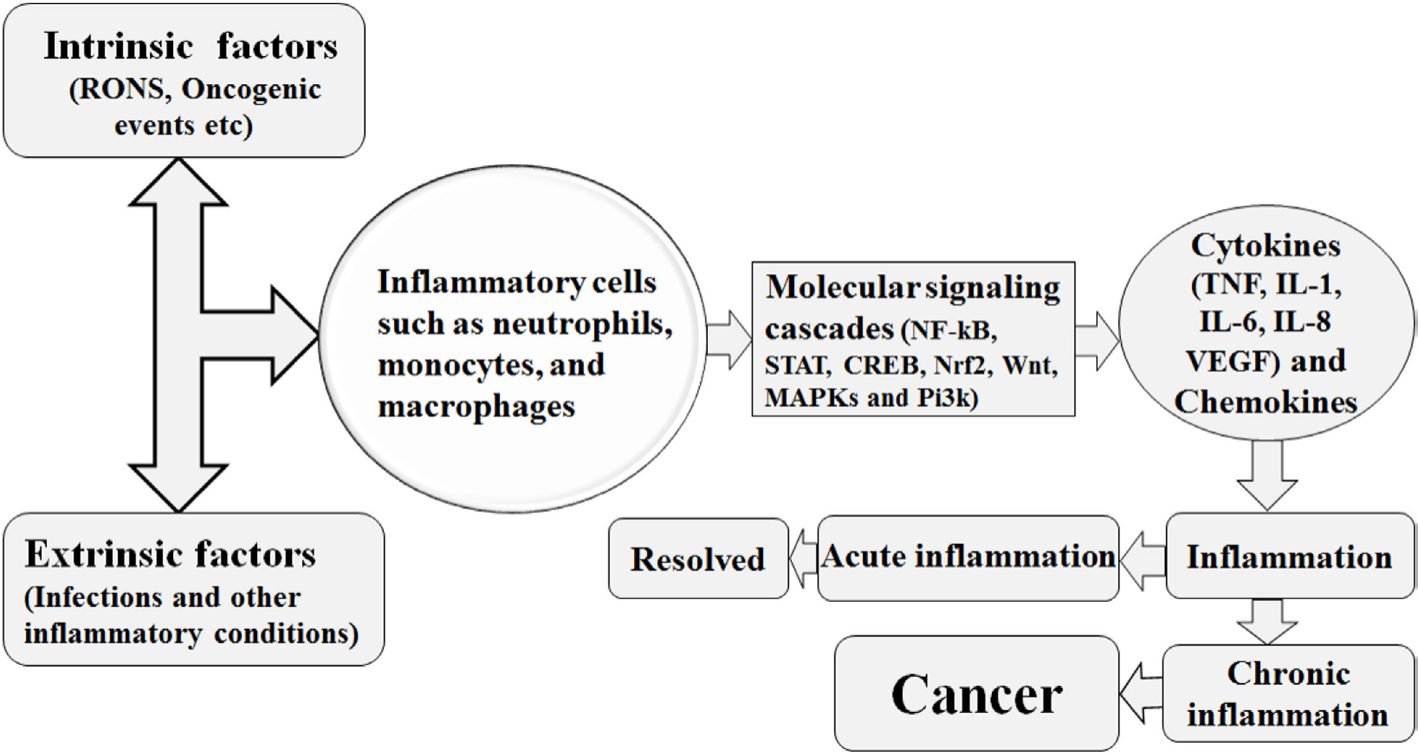
Various intrinsic and extrinsic factors trigger the recruitment of the inflammatory cells to the site of inflammatory insult, with resultant activation of several molecular signaling cascades. This signaling cascades are associated with increased production of inflammatory cytokines and the hence the establishment of inflammation. This inflammation is either of acute nature and resolved or is of chronic nature. The chronic form of inflammation is associated with cancer initiation.

During inflammation, the infiltration of immune cells such as neutrophils, macrophages, and eosinophils is associated with the generation of superoxide $\left(•O_{2}^{-}\right)$, nitric oxide (•NO), hydrogen peroxide (H_2_O_2_), hydroxyl radical (•OH), peroxynitrite (ONOO^−^), and hydrochlorous acid (HOCl•) to eliminate the pathogens and biological insult and these reactive species are called as RNOS (29). However, the constant presence of injurious stimuli is associated with continuous expression of these reactive species which tends to interact with the DNA, resulting in permanent genomic alterations such as point mutations, deletions, or rearrangements (29). Similarly, the RNOS synthesis facilitated by chronic inflammation, being highly reactive, interacts with DNA, protein, and lipid components of the cells and enhances cell transformation into malignancy (30). In addition, the DNA damage response in human gastric cancer was shown to be elevated by chronic inflammation (31). Investigation of chronic inflammation, DNA damage, and oncogenesis in human esophageal tissue revealed a direct relationship between chronic inflammation-mediated DNA damage and tumor transformation (31). Biopsies of 109 esophageal tissues stained with anti-8-OHdG antibody and immunostaining demonstrated that tissue with chronic inflammation was stained more densely than the non-inflamed tissue (31). Thus, from the above discussion, it is suggested that greater DNA damage is conferred by the RNOS in a chronic inflammatory microenvironment (31). The predominant macrophages and other leukocytes in inflammatory tissue generate RNOS to cope with the microbial invasion (4). Prolonged inflammation may be oncogenic by causing genomic instability and enhancing cellular proliferation and angiogenesis as described in Figure 1 (32, 33).

Patients with chronic IBD, including Crohn’s disease and ulcerative colitis, have shown increased progression toward cancer and were reported that the probability of developing CRC (colorectal cancer) 10 years after diagnosis with IBD was 2%, reaching the level of 8% after 20 years and 18% after 30 years (34, 35). Similarly, chronic inflammatory environment can drive tumor progression, because such a hostile environment is associated with increased growth factor secretion by inflammatory cells, and such proliferating cells are more prone to acquire genetic mutation and DNA damage, with resultant cell progression toward cancer (36). In addition, tumor necrosis factor-α (TNF-α) and migration inhibitory factor synthesized by inflammatory cells further exacerbate DNA damage (4). Immune cells infiltrate the tumor microenvironment, implicated in both early and advance cancer stages, and are involved in cross talk with the metastasizing cells. Correspondingly, the immune cells and their cytokines influence various stages of the metastatic process. Epithelial–mesenchymal transition, a critical stage in the metastatic process, is influenced by various cytokines such as transforming growth factor-β (TGF-β), IL-1β, IL-6, and TNF-α, which consequently stimulate NF-κB and signal transduction and activation of transcription (STAT3) inflammatory cascades. Similarly, the synthesis and activity of several proteases are enhanced by inflammatory mediators that are involved in tissue and extracellular matrix destruction, subsequently paving the way for tumor invasion. TNF-α and chemokines increase vascular permeability and promote the extravasation and migration of tumor cells (as shown in Figure 1) (37).

Cancer development is a multistep process characterized by self-sufficient and uncontrolled growth and proliferation, evasion from apoptosis and the immune system, increased angiogenesis, unresponsiveness to the effect of anti-growth signals, and the ability to metastasize to distinct sites (4). Epidemiological studies have reported that approximately 25% of cancer cases are related to chronic inflammation (38).

## Major Signaling Pathways and their Interplay in Cancer, Inflammation, and Immunity

Various molecular signaling pathways are involved in inflammation-mediated cancer and immune activation, and therapeutic approaches targeting these pathways are discussed below.

## Nuclear Factor-κB

Nuclear factor kappa B activation is central to both inflammation and oncogenesis, since NF-κB influences the expression of various genes related to malignancy and inflammation (39). NF-κB comprises a family of conserved and structurally related proteins including RelA/P65, Rel/cRel, RelB, NF-κB1/p50, and NF-B2/p52 proteins. NF-κB is kept inactive in the cytoplasm by inhibitory κB (iκB; IκBα, IκBβ, and IκBε), and to execute its functions it needs to be free from the inhibitory influence of iκB. All heterodimeric NF-κB complexes enhance the process of transcription; however, the homodimeric units, p50/50 and p52/52, suppress the process of transcription (40). Mitogen-activated protein kinases (MAPKs) and NF-κB signaling cascades are well-known classical pathways that regulate various cellular processes. NF-κB, being the hub of many signaling pathways, activates the expression of various genes and hence their products; for example, inducible cyclooxygenase (COX) and inducible nitric oxide produce prostanoids and nitric oxide (NO), respectively (41). Furthermore, NF-κB increases the expression of IL-2, a pro-inflammatory mediator, which amplifies T cell proliferation and differentiation. NF-κB activation in B-lymphocytes not only induces isotype class switching but also converts these cells into antibody-producing mature plasma cells (41).

**Figure 2:**
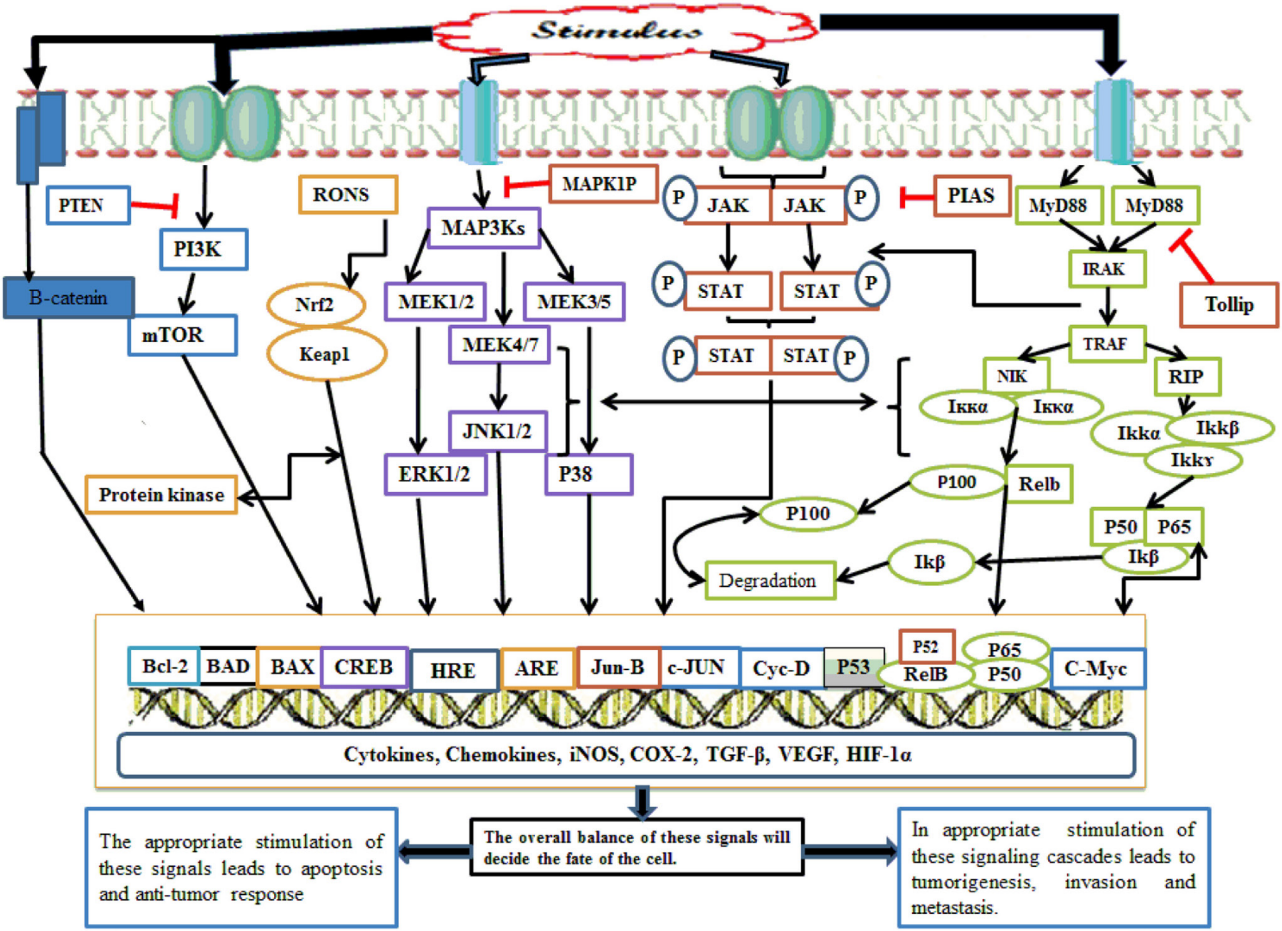
Whenever there is any inflammatory stimulus to the cell, there is activation of several molecular cascades such as NF-κB, Nrf2, MAPKinase, JAK/STAT, p53, PI3K/mTOR, CREB, and Wnt/Beta catenin. The appropriate activation of these molecular cascades is associated with the resolution of inflammation; however, inappropriate activation of such pathway causes persistent and chronic inflammation followed by tumor initiation, progression, invasion, and metastasis. NF-κB, nuclear factor-κB; Nrf2, nuclear factor erythroid 2-related factor 2; MAPKinase, mitogen-activated protein kinases; JAK/STAT, Janus kinases/signal transducing and activation of transcription; p53, transformation-related protein 53; PI3K/mTOR, phosphoinositide-3-kinase; CREB, cyclic AMP response element binding protein.

Nuclear factor kappa B also acts at the promoter region of cyclin D1 and amplifies its expression. Cyclin D1 increases cell division, and under its influence, favors the cell cycle progression from G1 phase to S phase (42). NF-κB makes the cell more resistant to apoptosis and necrosis by amplification of c-Jun-N-terminal kinase (JNK); this gene not only inhibits apoptosis protein 1 and 2 but also inhibits caretaker gene *P53*. This signaling pathway also promotes tumor metastasis *via* increasing the expression of chemokine 4 receptor receptors (5). The lipopolysaccharide (LPS)-mediated NF-κB signaling cascade follows two pathways: canonical and non-canonical/alternate. NF-κB is inactive in the cytosol until and unless it is complexed with inhibitory iκB. In the canonical pathway of NF-κB activation, the IKK complex having catalytic kinase subunits (IKKα and IKKβ) and scaffolding regulatory non-enzymatic protein IKKγ, also known as NF-κB essential modulator (NEMO), phosphorylates iκB. Thus, phosphorylated iκB undergoes subsequent proteasomal degradation. In the canonical pathway, Rel-A, c-Rel, RelB, and P50 NF-κB dimers are activated following TRAF recruitment by toll-like receptors (TLRs) and IKK complex-mediated iκB phosphorylation. This pathway is also regarded as the classical pathway and is the most vital pathway of NF-κB activation (43). Although the alternative pathway utilizes an IKK complex consisting of two IKKα units, NEMO is not involved. TRAF recruits NF-κB-inducing kinase (NIK), which phosphorylates and induces the IKKα complex. The IKK complex then activates the P52/Rel heterodimer after processing and cleavage following P100 phosphorylation (43). Alternative pathway involvement in carcinogenesis has been focused on in recent years, and its dysfunction has been implicated in several cancers. TRAF3 and NIK mutation in multiple myeloma (MM) resulted in NF-κB constitutive activation (44). Similarly, alternative pathway involvement in mammary carcinogenesis has also been documented (43). NF-κB activation is also influenced by Tir8 (also known as single immunoglobulin IL-1 receptors-related molecule), a member of the IL-1R family expressed in intestine (45). Tir8 negatively regulates the IL-1R/TLR complex signaling pathway, and this inhibition may be caused by possible trapping of IRAK-1 and TRAF-6 (Figure 2) (45).

## Nuclear Factor Erythroid 2-Related Factor 2 (Nrf2) Pathway

Cells and tissues are constantly exposed to oxidative stress generated inside and outside of the body, posing a threat to the integrity of the genome, and membrane lipids and proteins (46). This oxidative stress comprises reactive oxygen species (ROS) such as hydrogen peroxide (H_2_O_2_) and superoxides, and nitrogen species such as NO. ROS are neutralized by glutathione peroxidase, catalases, and peroxiredoxins (46). Moreover, protein-based cell defenses are also present, of which the expression is elevated in an oxidative hostile state. Among them, Nrf2 is a primary cell inducible transcriptional factor, present in many tissues of the body (46).

Nuclear factor erythroid 2-related factor 2 is kept inactive in the cytosol by Kelch-like erythroid cell-derived protein with CNC homology-associated protein 1 (Keap 1) (47). Upon stimulation, Nrf2 is liberated from the inhibitory effect of Keap 1 and translocates into the nucleus, where it interacts with *cis*-acting DNA enhancer antioxidant response elements (AREs) (48). Nrf2, after dissociation from Keap 1, forms a complex with Maf, a small protein family, to activate AREs (49). Initially, it was identified that Nrf2 induces phase 2 enzymes and antioxidant molecules that inactivate oncogenic reactive species and thus exert an antitumor effect (Figure 2) (50). Keap1–Nrf2 dysregulation *via* mutation in either Nrf2 or its inhibitor Keap1 has been associated with the initiation of various tumors, including adenocarcinoma, and gall bladder and liver cancers. Keap1 mutation stabilizes Nrf2, which in turn, causes increased synthesis of cytoprotective antioxidant proteins in normal body cells and transformed cells. Nrf2 overexpression secondary to Keap1 dysfunction, on the one hand, protects the normal cells from carcinogenic reactive substances, but on the other hand, promotes cancer by protecting the cells from anticancer drugs, especially those acting by generating fatal oxidative molecules; thus, the Keap1–Nrf2 pathway acts as a “double-edged sword” (51).

## MAPK Pathway

Mitogen-activated protein kinase activation is concerned with cell growth, differentiation, survival, and immune and stress responses (52). MAPKs exhibit a unique character in the sense that they are phosphorylated at three stages, from upstream to downstream, and each step is regulated by different kinases, hence known as “core signaling modules.” In the initial step of phosphorylation, MAP3Ks/MAPK kinase kinases (MAPKKKs) govern the phosphorylation of MAPKKs (MAP2Ks/MAPK kinase) at serine/threonine amino acid residues. Once phosphorylated, the MAP2Ks phosphorylate the MAPKs. MAP2Ks and MAPKs have few kinases, while MAPKKKs comprise many kinase protein families. Three MAPK-mediated pathways are well studied, including extracellular signal-regulated kinase (ERK1/2), and this pathway is triggered by cytokines, growth factors, hormones, and osmotic stress (53). After activation, ERK1/2 then translocates into the nucleus and phosphorylates and activates mitogen-activated protein kinase activated protein kinase, and consequently increases cell growth and proliferation (54). The P38 MAPK activation is under the influence of inflammatory cytokines, stress, pathogen-associated molecular patterns (PAMPs), and damage-associated molecular patterns (DAMPs). MKK3, MKK4, and MKK6 phosphorylate and hence activate p38 MPAKs. MAP3Ks involved in p38 activation include MAPK/ERK kinase kinase 3/4, TGF-β-activated kinase-1 (TAK1), mixed lineage kinase 3, tumor progression locus 2 (TPL2), thousand-and-one kinases 1/2 (TAO1/2), leucin zipper and sterile-α motif kinase 1, dual leucine zipper kinase (DLK), and apoptosis signal-regulating kinase 1/2 (ASK1/2). The activated p38 is dephosphorylated by MPK1 phosphatase, having high specificity for p38; however, other phosphatases also dephosphorylate p38 such as MPK2/4 and MPK5/7 (54). JNK activation is triggered by genotoxins, inflammatory cytokines, protein synthesis inhibitors, mitogens, DAMPs, and PAMPs. JNK is phosphorylated and activated by various types of MAP2Ks including MKK4 and MKK7. However, MAP2Ks are phosphorylated upstream by various isoforms of MP3Ks including TPL2, TAK1, ASK1/2, DLK, TAO1/2, and MLK1/2/3. Once activated, JNK is shuttled into the nucleus to regulate the expression of genes involved in growth and proliferation, such as c-Myc, c-Jun, Jun-B, EIK, P53, and NFAT. The role of JNK has also been elucidated in inflammatory cytokine production, tumorigenesis, and insulin resistance (Figure 2) (54).

## Transformation-Related Protein 53/p53

Cells under stressful conditions increase *P53* expression to generate ROS for cell apoptosis, whereas in normal physiological conditions, *P53* expression reduces ROS production and protects the cell from the deleterious effects of ROS, thus acting as a double-edged sword (55). By inhibiting ROS and inducing the synthesis of TGF-β, *P53* acts as tumor-suppressor gene (56). *P53* is subjected to very strict control by inhibitory molecules including mouse double minute 2 homolog (MDM2) and MDMX, also known as MDM4 (57). *P53* activation is directed by specific stress signals in a tissue-specific manner and undergoes extensive posttranslational modifications such as acetylation, phosphorylation, neddylation, and monoubiquitination. This signal-specific *P53* modification activates different sets of genes, such as expression of Puma genes (*P53*-upregulated modulator of apoptosis), Bax (Bcl-2 associated x-protein), Noxa, and Apaf-1 (apoptotic protease activating factor-1) to induce apoptosis. Activation of 14-3-3δ, cyclin-dependent kinase inhibitor 1, Cdc25c, and P21/WAF1, results in reversible cell cycle arrest, while induction of Pai-1 (plasminogen activator inhibitor type 1) and P21 causes complete cell cycle arrest (57). A National Institute of Health study reported that 61% of bladder cancer cases showed *P53* mutation, and its mutation was associated with invasive types of tumors (58). Several studies demonstrated that *P53* nullifies the activation of the major inflammatory NF-κB signaling cascade by competing with cyclic AMP response element binding protein (CREB) and P300 coactivator protein. *P53* directly inhibits the P65 NF-κB unit, and indirectly suppresses the IKKα-mediated activation of NF-κB. This *P53*-mediated NF-κB suppression inhibits the synthesis of pro-inflammatory mediators such as COX-2, IL-6, and iNOS (Figure 2) (59).

## Janus Kinases (JAK)/Signal Transducing and Activation of Transcription (STAT) Pathway

The STAT pathway is linked upstream with the family of protein kinases known as JAKs. Cytokines interact with their cognate surface receptor and receptor-associated JAK molecules, which cross-phosphorylate each other at tyrosine residues. The phosphorylated JAK molecules recruit STAT protein *via* their SH2 domain and undergo phosphorylation and activation. Following phosphorylation, the STAT proteins are shuttled into the nucleus either in homo or heteromeric form to regulate the transcription of various genes (60). The STAT signaling pathway is finely tuned at various steps. Protein inhibitors of activated STATs inhibit STAT activity *via* interfering with its DNA binding domain. Another protein, small ubiquitin-like modifier E_2_ ligase, increases STAT protein degradation and hinders its activation (61).

Despite being under such tight regulation, this signaling pathway is subjected to mutations. STAT5b somatic mutation is found in lymphoid malignancies (62); however, STAT5 also exerts an antitumor effect. In NK cells, STAT5 inhibits the production of pro-tumorigenic and angiogenic factor VEGF-A (63). IL-6 in prostate gland epithelial cells interacts with its receptor to form an IL-6R gp130 protein hexameric complex, causing downstream activation of the JAK2/STAT pathway. JAK2 gain-of-function mutation has been observed in thrombocytopenia, polycythemia vera, and myelofibrosis neoplasia (61). The JAK/STAT pathway is dephosphorylated and downregulated by protein tyrosine phosphatases (PTPs) such as PTP1B (PTPN1) and TC-PTP (PTPN2). PTPs have a total of 107 genes in humans, which are divided into 4 sub-classes: class I, class II, class III, and class IV; however, class I and class III are involved in JAK/STAT pathway regulation. PTP1B exerts anti-inflammatory activity, and it is reported that mice lacking PTPB1 are susceptible to developing a chronic inflammatory state. In contrast with this, PTP1B counteracts IL-10-mediated STAT3 downstream anti-inflammatory responses. PTP1B has demonstrated both tumorigenic and antitumor behavior in various studies, since mice lacking PTP1B were tumor resistant; however, PTP1B deficiency has also been observed in colon, breast, gastric, and prostate tumor development. Mice with deficiency of CT-PTP (PTPN2) within 1–2 weeks of birth not only developed an inflammatory state, characterized by mononuclear infiltrates, but also showed increased synthesis of TNF-α, IFN-γ, and NO. Six percent of T cell lymphoblastic leukemia cases show deficiency of the PTPN2 gene; however, its overexpression has also been reported in B-cell lymphoma (Figure 2) (60).

## Phosphoinositide-3-Kinase (PI3K) Pathway

Phosphoinositide-3-kinase receives signals upstream from G protein-coupled receptors and receptor tyrosine kinases (64). PI3K acts upon phosphatidylinositol-4,5-biphosphate substrate and phosphorylates it to phosphatidylinositol-3,4,5-triphosphate (PIP_3_), and the reaction is negatively regulated by phosphatase and tensin homolog (PTEN) tumor suppressor phosphatase, SHIP1/2, and INPP4B. The PIP_3_ product of the PI3K pathway acts on PREX1/2, Akt, and 3-phosphoinositide-dependent protein kinase 1 (PDK1) members of pleckstrin homology domain-containing proteins. Akt undergoes phosphorylation-dependent activation by PDK1 at threonine 308 residues and mTORC2 complex at serine 473. Once activated, Akt triggers downstream activation of numerous substrate molecules concerned with cell growth, protein synthesis, survival, and metabolism (Figure 2) (65). PI3K mutations have been observed in various tumors, and its alteration may take place at several steps including gain-of-function mutations in genes encoding the P110α catalytic unit and rarely the P110β catalytic unit. The P85α regulatory subunit and loss-of-function mutations in PI3K inhibitor phosphatases such as PTEN and INDP4B are implicated in various tumors (65). PTEN loss-of-function mutation followed by increased activity of PI3K elevates programmed death ligand 1 (PD-1), an immune cell suppressant factor, whereas PI3K inhibitors increase the immune cell response to tumors (66). Patients with pancreatic cancer show the lowest 5-year survival rate among all other cancers, which is initiated by various genetic alterations; however, K-Ras is the most commonly implicated gene in pancreatic adenocarcinoma, with a 70–90% contribution. The K-Ras family of proteins activate the PI3K/Akt signaling cascades downstream, and this Akt downstream activation is thought to be the main cause of worse prognosis with pancreatic tumors (67).

## CREB Signaling Pathway

Cyclic AMP response element binding protein family members, when activated, bind to cAMP response elements (CRE), and promote the recruitment of coactivator proteins such as CBP/p300, thereby initiating transcriptional machinery and inducing CREB target genes (68–74). The role of CREB in cell survival has also been described in a number of tissues, such as nerve growth factor (NGF)-induced phosphorylation of CREB, and it was proposed that phosphorylated CREB induces genes that confer specificity to NGF signals that are associated with increased survival and differentiation of neurons. Furthermore, CREB-mediated gene expression is essential for NGF-dependent cell survival and is critical to promote survival of sympathomimetic neurons (68–74). Bcl-2 is also activated by NGF in a CREB-dependent manner, and this Bcl-2 expression is associated with increased cell survival. Thus, it is apparent that activation of CREB induces the survival of neurons *via* activating downstream target genes that encode prosurvival factors (68–74). Recently, numerous reports have revealed a significant emerging role of CREB in various cancers. For example, patients with acute lymphoid leukemia or acute myeloid leukemia show increased CREB expression in samples of their bone marrow, and this CREB expression is associated with worse prognosis in patients with AML (68–74). Overexpression of CREB promotes increased survival in myeloid cells, while CREB downregulation tends to decrease cell proliferation and survival. One-fourth of CRE-containing sequences regulate cellular metabolism. In liver, CREB regulates the process of gluconeogenesis *via* the phosphoenolpyruvate carboxylase enzyme. Similarly, enzymes such as pyruvate carboxylase, ornithine decarboxylase, and lactate dehydrogenase also contain CRE sequences in their promoter region (68–74). CREB involvement in cancer was first implicated in clear-cell sarcomas of soft tissues (CSSTs). Most CSSTs contain a chromosomal translocation that fuses the DNA binding and bZip domains of ATF1 to the Ewing’s sarcomas gene *EWS*. This Ewing gene and ATF1 protein interaction leads to stimulation of proliferation and suppresses the apoptotic process. More recently, a EWS–CREB fusion protein was implicated in a subset of patients with CSST. EWS–ATF1 and EWS–CREB fusion proteins are constitutively stimulated and promote the expression of CREB target genes independently of a growth signal stimulus (Figure 2) (68–74).

## Wnt/Beta Catenin Pathway

The Wnt signaling pathway has an important contribution to embryonic development and homeostasis of mature tissues. The role of this pathway is also implicated in cell differentiation, controlling cell proliferation, apoptosis, polarity, and migration (75–80). In the absence of specific Wnt ligands, the β-catenin located within the cytosol is destroyed by the destruction complex composed of three proteins, such as antigen-presenting cell (APC), AXIN, and GSK3B, following phosphorylation. Wnt protein induces the expression of the intracellular pathway after interaction with the FZD seven-pass receptor and its single-pass transmembrane coreceptors such as low-density lipoprotein receptor-related protein 5/6 (LRP5/6) or receptor tyrosine kinase-like orphan receptor 2 (75–80). Wnt protein binds the FZD receptor at its cysteine-rich domain and triggers the phosphorylation of LRP5/6, with subsequent formation of a FZD–LRP5/6 heterodimeric complex. This phosphorylated LRP5/6 facilitates the recruitment of the Axin–GSK3–CK1 complex to the Wnt–LRP5/6–Dv1 complex at the cell membrane (75–80). Recruitment of the Axin–GSK3–CK1 complex inhibits the interaction between this complex and APC and β-catenin to form the destruction complex. As a result, the level of β-catenin will be increased in the cytosol, and this surge will enhance the translocation of β-catenin into the nucleus (75–80). Following translocation into the nucleus, β-catenin binds to the N-terminal T-cell factor/lymphoid enhancing factor and induces the expression of Wnt-directed genes involved in cellular development and maintenance (75–80).

The Wnt signaling pathway is regulated by several means, such as by two serine/threonine phosphatases: protein phosphatase 1 (PP1), and protein phosphatase 2A (PP2A) (75–80). These phosphatases bind Axin, APC, GSK3, and CK1 to inhibit their interaction with β-catenin. PP1 enhances the dephosphorylation of Axin and leads to the disruption of the GSK3–Axin interaction, and ultimately inhibits the formation/disruption of the multi-protein destruction complex, whereas PP2A dephosphorylates β-catenin directly (75–80). However, despite being subjected to such tight regulation, alterations in genes encoding various components of Wnt/B-catenin signaling such as Wnt, Fzd, APC, and LRP5/6 have been reported in numerous studies. Alterations in genes encoding APC have been described in sporadic colorectal cancer development. On the other hand, alteration in the porcupine enzyme-encoding gene, which resides in the endoplasmic reticulum (ER) and facilitates the process of Wnt secretion, has also been demonstrated in focal dermal hypoplasia (75–80). Canonical Wnt pathway involvement in head and neck squamous cell carcinoma (HNSCC) has also been reported in various studies. Evidence for this association has been provided by cDNA arrays on a patient sample, which showed that expression of Fzd, Fzd homolog 3, and Dv1 homolog genes, key components of the Wnt signaling pathway, were twofold to fivefold raised in patients with HNSCC compared with normal patient tissue (75–80). Similarly, increased expression of Fz has also been observed in gastrointestinal carcinomas. This Fz upregulation is associated with increased interaction of Wnt-Fz and has been demonstrated in several GIT carcinomas (Figure 2) (75–80).

## Innate and Adaptive Immunity: Inflammation and Cancer

The innate immune system confronts pathogens *via* a large number of diverse pathogen-recognition receptors (PRRs) and by the generation of nonselective and random antigen-specific receptors molecules (81). The innate immune system comprises mucosal epithelial barriers, immune molecules, and immune cells. Innate immunity is without memory and is non-specific in nature and is involved in the presentation of antigens to the adaptive immune cells (81). The innate immune system generally recognizes the PAMPs expressed by the pathogen *via* PRRs and induces the activation of transcriptional factors including NF-κB (81). PRRs comprise nucleotide-binding domain-like receptors (NLRs), TLRs, and retinoid inducible gene-1-like receptors, which are involved in PAMP recognition. PAMPs induce innate immune responses and increase the expression of inflammatory cytokines such as IFNs, ILs, and TNFs. TLRs are vital members of the PRR family, expressed by numerous cells of the body. TLRs mediate the induction of downstream signaling cascades in dendritic cells (DCs) such as NF-κB *via* the MyD88 pathway followed by activation of the acquired immune system. The NLR component of the PRRs resides in the cytoplasm and is concerned with the sensing of internal signals. The NLR and TLR pathways overlap when activated by their ligands, such as LPS (82–84). NOD proteins NOD1 (CARD4) and NOD2 (CARD15) are regarded as the first reported PRRs located intracellularly for the recognition of PAMPs. The NLR family is also known as NATCH–LRR, NOD–LRR, and CATERPILLAR and is involved in various host immune responses (82–84). NLRs composed of three well-distinguished domains: pyrin (PYD) effector domain, or amino-terminal CARD (caspase recruiting domain), of which NAIP and NOD5 are exceptional cases in which these domains are absent; NACHT domain or nucleotide-binding and oligomerization domain; and carboxy-terminal LLRs with varying numbers (82–84). NLR proteins are divided into two major sub-classes, the NOD proteins having 5 members including NOD1, NOD2, NOD3, NOD4, and NOD5 (they have a CARD effector domain) and 14 members of the NALP clan (they have a PYD effector domain in contrast to a CARD domain) (82–84). Of the two CARD-containing domains, CIITA, IPAF, and a BIR-containing (baculovirus IAP repeat) NAIP domain comprise 22 members of the NLR protein family that are known to date. NOD1 and NOD2 proteins recognize bacterial peptidoglycan-derived components such as meso-diaminopimelic acid (DAP) and muramyl dipeptide (MDP), respectively (82–84). Following recognition of DAP and MDP, NOD2 recruits RIP2, which in turn activates MAPKs and the NF-κB pathway (82–84). The mechanism of MDP entry into the cytoplasm is still unknown. Furthermore, it is also unknown how MDP is recognized by NOD2; however, the LRR domain is necessary for MDP-mediated signaling (82–84).

Acquired immunity is more specific compared with innate immunity and possesses memory immune cells, which upon subsequent exposure to the same antigen, result in robust activation of the immune machinery (84). T cells, after encounter with pathogens, are clonally expanded and are differentiated into various subsets, such as Th1, Th2, Th17, and Treg cells (85). Th1 cells are active against intracellular bacteria, whereas Th2 cells are involved in allergic reaction and also confer protection against parasitic infections (85). Th17 cells respond to extracellular bacteria and fungi, whereas Treg cells aid in tissue repair and remodeling. However, inappropriate activation of T cells is also implicated in various inflammatory conditions by producing excessive pro-inflammatory cytokines (86). The various cells involved in innate immunity include macrophages, neutrophils, and DCs. Both components of the immune system, innate and adaptive, augment the effect of each other to resist and protect the body against the invading microorganism (83).

## Role of Various Immune Cells in Inflammation and Cancer

Various immune cells partake in tumor immunity either to inhibit or promote cancer initiation (87). However, the exact role of immune cells in tumor immunity is yet to be determined. In tumor immunity, effectors cells such as NK cells, and CD4^+^ and CD8^+^ cells are in competition with Treg cells, because Treg cells strongly inhibit effector cells that mediate antitumor immunity (87). T lymphocyte penetration into the tumor microenvironment is found to have a significant impact on tumor suppression and an improved patient condition. However, tumors still grow and survive despite lymphocyte infiltration into the tumor microenvironment (88). Following the activation and maturation of T cells into either CD4^+^ T helper or CD8^+^ cytotoxic T cells, they play a critical role in inflammation and tumorigenesis. Similarly, the stimulation of CD4^+^ helper and CD8^+^ cytotoxic T cells by immunotherapy has shown encouraging results in cancer therapy (89). CD4^+^ T helper cells and CD8^+^ T cells that synthesize INF-γ cytokines are believed to exert an antitumor effect by killing tumor cells and restricting tumor growth (28). The presence of INF-γ cytokines at the tumor microenvironment has a positive impact on tumor prognosis (28). However, the role of T cells has also been implicated in the development of gastric cancer secondary to *H. pylori* infection (90–92).

Macrophages are a primary component of the innate immune system and the first cells to initiate inflammation after engulfing foreign product; thus, they are utilized in many *in vitro* inflammatory studies (93). Macrophages predominate in chronic inflammation and reside in the tissue, after circulating monocytes leave the circulation and become part of the tissue macrophages. Macrophages exist in two polarized states: M1 polarized macrophages produce pro-inflammatory cell-mediated responses, whereas M2 polarized macrophages play a role in immune suppression, tissue regeneration, and wound healing (94). Macrophages are involved in phagocytosis, cellular and microbial product degradation, and apoptosis (95). M2 macrophages synthesize IL-10 anti-inflammatory cytokines at a higher concentration when compared with M1 macrophages, thus maintaining a proper and balanced immune response (96). Neutrophils, also known as polymorphonuclear leukocytes, contribute at the highest concentration to the leukocyte pool. Previously, neutrophils were only known to be involved in anti-microbial activity. However, diverse functions of neutrophils have subsequently been identified, including regulation of innate and acquired immunity and its promising role in tumor immunity (97). TANs are shown to be involved in primary tumor progression; however, inhibition of the TGF-β signal recruits antitumor TAN cells. Similarly, neutrophils in patients with cancer prevented tumor metastasis by eradicating the metastatic cancer cells (98). The contribution of TANs to tumor development ranges from its initiation, invasion, and metastasis to angiogenesis. TANs have a negative impact on various tumors in patients, such as renal cancer, glial cell cancer, melanoma, hepatic carcinoma, colon tumor, and gastric and cervical cancers (99). In the case of the immune response, both neutrophils and macrophages act together against tumors. TANs and tumor-associated macrophages in the tumor microenvironment show a significant plastic nature. Dysfunctional macrophages at the tumor site attract neutrophils to initiate and sustain the process of angiogenesis and tumor progression in a paracrine fashion (100). Different studies reported that a high number of neutrophils and a high neutrophil/lymphocyte ratio (NLR) are related to poor outcome. Patients with T4 head and neck cancer exhibit a higher neutrophil count than patients with T1 and T2 types (101). A low concentration of TANs was associated with a better 5-year survival in stages III and IV cancer patients than a higher concentration of TANs in patients with squamous cell carcinoma. Similarly, high NLR has been correlated with poor cell differentiation, advanced stage tumors, worse prognosis, metastasis, and relapses (102).

## Mediators: Cancer, Inflammation, and the Immune System

Cytokines are key signaling molecules of inflammation and the immune system. Cytokines are synthesized in response to an altered homeostatic environment and take part in diverse cellular functions. They are usually classified into two classes: anti-inflammatory cytokines, such as IL-4, IL-10, IL-13, IFN-α, and TGF-β, and pro-inflammatory cytokines, such as IL-1β, IL-6, IL-15, IL-17, IL-23, and TNF-α (103). TGF-β highly influences various functions of the immune system. It has a plethora of effects on the cells of the acquired immune system, especially on CD4^+^ T cell effectors and regulatory responses (104). In normal embryological body development, TGF-β induces potent apoptosis (105). However, it also regulates apoptotic, angiogenic, immunogenic, and anti-tumorigenic responses in the adult (106). Despite its pro-apoptotic role in the initial tumor setting, a controversial tumorigenic role of TGF-β has been reported in the late phase, owing to cell resistance to its inhibitory signals (107). TGF-β negatively affects the activity of various immune cells including NK cells. It suppresses NK cell growth and activity and induces the synthesis of IFN-γ (108). Although TGF-β suppresses the growth of normal as well as transformed cells, in pancreatic ductal adenocarcinoma, it is tumorigenic by promoting angiogenesis and immune suppression, and also by rendering chemoresistance and facilitating invasion and metastasis (109). Similarly, TNF-α is considered as an anti-tumorigenic factor at high concentration (110); however, at moderate concentration, TNF*-*α can stimulate angiogenesis, metastasis, and cause damage to the DNA as shown in animal models (111). TNF*-*α is mostly synthesized by macrophages as well as by cancerous cells in small amounts (110). Several studies have reported that the pro-tumorigenic effect of TNF may be mediated by inhibiting DNA proofreading and DNA repair mechanisms (4). The IL-1β family of cytokines consists of several members and is implicated in the growth and development of several human tumors including esophageal, gastric, colorectal, and ovarian cancers (112). IL-1 together with TNF*-*α are alarming cytokines, secreted by various immune cells. IL-1β potentiates its own as well as the production of other pro-inflammatory factors including NO, COX-2, chemokines, cytokines, and metalloproteinases (MMPs) (113). IL-1 produced under the influence of leptin indirectly increases the expression of VEGF/VEGFR-2. This triad of cross talk between leptin, IL-1, and VEGF increases growth and survival in breast tumors and promotes angiogenesis (114). IL-2 is synthesized mainly by T helper cells (115). At high doses, IL-2 activates CD8^+^ cytotoxic T cells and is thus approved for the management of various tumors (115). IL-2 is an important factor in promoting the growth and stimulation of CD8^+^ T cells and NK cells. In a study of CD8^+^ T cell differentiation into long-lived memory precursor effector cells (MPECs), T cells expressing CD127^hi^ and KLRG1^lo^ and short-lived effector cells (SLECs) expressing KLRG^hi^ and CD127^lo^ are regulated by B-lymphocyte-induced maturation protein 1 (Blimp-1). However, IL-2 increases Blimp-1 expression in CD8^+^ T cells. This Blimp-1 expression favors the differentiation of CD8^+^ T cells into SLECs during acute infection, whereas Blimp-1-deficient CD8^+^ T cells are differentiated into long-lived MPECs (116).

Among, IL-6 is a pro-tumorigenic cytokine by engaging with the K-Ras oncogene in pancreatic and lung cancers. Lung adenocarcinoma shows an increased level of IL-6 and was related to poor patient quality of life (117). High serum levels of IL-6 were observed in 37% of MM cases. In patients with increased levels of IL-6, MM behaves more aggressively, exhibiting a higher growth rate and worse prognosis (112). The pro-tumorigenic nature of IL-6 has been studied in various cancers including breast, pancreas, renal, and prostate tumors. Similarly, various researchers found an increased blood IL-6 level in individuals with colorectal cancer, while its concentration was directly correlated with colorectal cancer size, stage, metastatic state, and patient survival. Concordantly, IL-6 is a major contributor in IBD, mainly by acting on GIT immune cells, and activates mucosal immune cells and renders them more resistant to apoptosis. This resistance to apoptosis is conferred by activating anti-apoptotic genes such as Bcl-x and Bcl-2 (118). IL-6 performs various pro-inflammatory functions in different pathophysiological conditions including Crohn’s disease and rheumatoid arthritis (RA)-associated inflammation (119).

In contrast to other pro-inflammatory cytokines, IL-10 plays a critical role in the inflammatory response by restricting the synthesis of several pro-inflammatory mediators including TNF*-*α, IL-6, and IL-12. IL-10 hinders many immune cell functions including macrophage-mediated pro-inflammatory cytokines. Lack of IL-10 has been shown to predispose individuals to various pro-inflammatory conditions such as IBD, while exogenous IL-10 administration *in vitro* resolved the inflammation and also inhibited autoimmune responses (94). IL-10 synthesized by anti-inflammatory macrophages inhibits the cardinal anti-inflammatory signaling pathway of NF-κB, thus limiting inflammation and facilitating tissue repair and remodeling (120).

**Figure 3:**
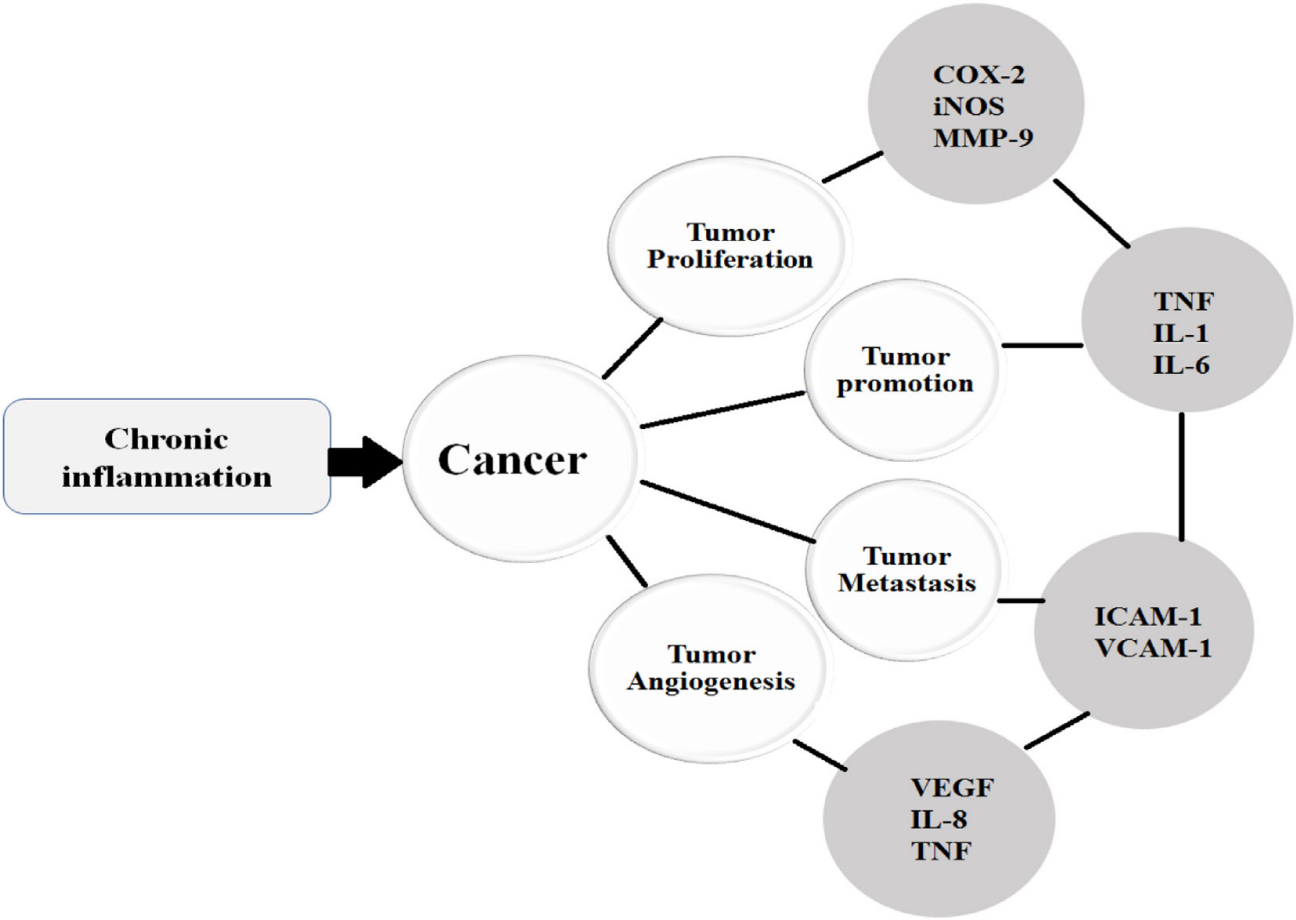
Once the chronic inflammatory microenvironment is established and trigger the initiation of cancer, then the cancer undergoes various stages such as proliferation, promotion, metastasis, and angiogenesis under the influence of various cytokines.

Similar to other cytokines, VEGF is also implicated in various tumorigenic conditions and regulates the process of angiogenesis. Endothelial cells divide only in disease states such as tumors, endometrial growth, and injury. Angiogenesis is triggered by various angiogenic factors including VEGF, EGF, TNF*-*α, and IL-8; however, VEGF is a critical angiogenic factor (121). Studies show that VEGF enhances cell proliferation, permeability, migration, and increases cell survival by inhibiting apoptosis. On the other hand, VEGF-targeted therapies have been shown to inhibit tumor growth, angiogenesis, and permeability (122). VEGF receptors are expressed by human colorectal cancer cells. One study showed that VEGF exhibits anti-apoptotic activity in colorectal carcinoma (CRC) cells *via* a novel intracrine pathway rather than by paracrine and autocrine canonical pathways. This novel intracrine signaling pathway has been exploited to treat VEGF-mediated angiogenesis (Figure 3) (123).

## Novel Immunotherapeutic Approaches toward Cancer

Immune therapeutic-based current approaches comprise vaccines, chimeric antigen receptor (CAR)-expressing T cells, immune checkpoint inhibitors, and bispecific antibodies. The basic aims of all these therapies are to amplify the “killing power” of various immune cells and to target the malignantly transformed cells (124).

## Immune Cell-Mediated Immunostimulatory Approaches toward Cancer

The basic focus of the immunotherapeutic approach is to amplify the immune response specifically against tumor cells (125). Various immune cells such as macrophages, neutrophils, and microglial cells have been tried for drug delivery to treat tumors. Similarly, T cells secreting IL-2 have been used to treat tumors in the past. Recently, adoptive cell transfer (ACT) has utilized tumor-infiltrating lymphocytes (TILs) obtained from the tumor microenvironment or from circulating peripheral T cells. ACT is infused back into lymphodepleted patients after *ex vivo* expansion (126). However, TILs expressing a T-cell receptor (TCR) population of a heterogeneous nature have been directed against various antigens. To overcome this problem and improve TIL tumor targeting, T cells are modified by genetic engineering to express α/β-TCR or CARs along with native TCRs. CARs have two domains, including an extracellular domain specific for antigen binding plus one or two intracellular T cell signaling domains (126). These artificial recombinant CARs cause the production of reactive T cells directed against the tumor antigen. Furthermore, these artificial CARs do not require MHC molecule and T cell interaction, instead acting directly against cells expressing tumor antigens. However, this therapy has certain limitations such as to produce CAR cells in higher amounts *ex vivo*, which may compromise its *in vivo* activity and endurance (126). Finally, α/β-TCR and CARs can also bind target protein on normal (non-tumor) cells and exhibit toxicity (126).

Furthermore, the current regimen using autologous ACT is quite time-consuming, expensive, and very difficult to obtain sufficient CAR-T cell with appropriate quality for the elder and newborn patients. Thus, to ensure the easy availability of CAR-T therapy, the development of allogeneic adoptive transfer approach is highly desirable, which utilizes the universal CAR-T obtained from the healthy donor derived T cells to exploit against multiple diseases (127). To make this approach workable, the αβ TCR on allogeneic CAR-T cells should be removed to inhibit the graft-versus-host responses and human leukocyte antigens class I (HLA-Is) should be eliminated to render their immunogenicity minimized. It was previously reported that TCRα subunit constant (TRAC) mutation trigger the loss of αβ TCR on T-cell surface and beta-2 microglobulin (B2M) is necessary for the HLA-I heterodimers expression on cell surface (127). Thus, to develop the most potent and universal CAR-T cells, multiple genes elimination will be required simultaneously as described. The multiple gene editing within CAR-T induced by (CRISPR) and CRISPR-associated protein 9 (Cas9) system (CRISPR-Cas9), efficiently leads to the development of TRAC and B2M, and was evaluated *in vivo* as well as *in vitro* and exhibited significant inhibitory activity against the lymphoma xenograft mouse model (127).

Multiple myeloma is the malignancy of mature B-cells, confined to bone marrow. In MM, the tumor microenvironment is infiltrated by various cells and their intercommunication leads to the amplification of MM cell growth and survival (128). In MM, type 1 NK cells were found to be functionless and unable to produce IFN-γ, despite their detectable level in the body. A study was conducted to evaluate the role of NK type 1 cells on the growth of MM cells. DCs loaded with α-GalCer were injected into patients with MM myeloma (two doses per month). The results demonstrated that the NK cell number was increased up to 100 times and the patient overall condition was improved. The MM cells expressed CD1d molecules at the early stage and were sensitive to the NK cells. However, as the tumor advanced, the CD1d expression was decreased and correlated with decreased survival and poor outcome. The monoclonal anti-CD1d antibodies directed against the CD1d *in vitro* induced apoptosis *via* increasing the expression of Bax pro-apoptotic genes (128). The murine study revealed that DCs loaded with α-GalCer significantly increased invariant NK cells synthesizing IFN-γ, while increasing the survival of MM mice by up to 1 week. Similarly, a vaccine composed of α-GalCer-loaded MOPC315BM myeloma cells significantly reduced tumor growth and proliferation. Furthermore, the vaccine also increased overall survival and upregulated antibody-mediated and cellular responses such as CD8^+^ T cytotoxic activity, induced memory cells, and suppressed Treg cells (129).

Dendritic cell-mediated immune activation offers several advantages, including a good safety profile and rare association with immune-related toxicities. DCs can be administered alone after chemotherapy or they can be loaded with various types of antigens such as peptides, proteins, whole tumor lysate, or genetic material delivering the desired antigen (electroporated DNA, RNA, or transduced virus) *ex vivo* before infusion into the patients. The use of whole cell as an immunotherapy offers an advantage that will present a range of tumor-associated antigens from that particular tumor to the immune system, therefore predicting a better immune response. DCs loaded with hypochlorous acid-oxidized whole tumor lysate (inducing primary necrosis and enhancing the immunogenicity of lysed tumor cells) show encouraging results in patients with ovarian cancer. The whole tumor cells that were tried for the treatment of ovarian cancer were autologous tumor cells electroporated with FANG vector, i.e., gemogenovatucel-T (FANG vaccine), plasmid-encoded granulocyte macrophage-colony stimulating factor (GM-CSF; a potent stimulator of DC maturation), and a bi-shRNA targeting furin convertase, thereby downregulating TGF-β1 and -β2 endogenous immunosuppressive growth factors (130).

## Cytokine-Mediated Immunostimulatory Approaches toward Cancer

Currently, research is in progress to target cancer by enhancing the immune system. The introduction of antibodies against immune checkpoint inhibitors such as PD-1 and cytotoxic T lymphocyte antigen-4 (CTLA-4) instead of targeting the tumor is a novel approach for cancer treatment (131). Similarly, the hypoxic and adenosine-rich “hypoxia-A2-adenosinergic” (A2AR) tumor microenvironment inhibits T cell and NK cell infiltration into the tumor microenvironment. In the tumor microenvironment, hypoxia induces the synthesis of hypoxic inducible factor-1α (HIF-1α). In turn, HIF-1α tends to convert ATP/ADP into adenosine extracellularly *via* CD73/CD39 enzymes. This adenosine, concentrated extracellularly, acts on A2AR and A2BR (hypoxia-A2-adenosinergic receptors) to induce the synthesis of immunosuppressive cAMP. A study reported that antagonists that target the A2AR and A2BR, on one hand, enhance the T cells effector function against the tumors, while on the other hand, also induce apoptosis in the cancerous cells and INF-γ, which further prevented angiogenesis (131). To overcome pathways of immune evasion, different strategies have been devised, particularly to target CTLA-4 and PD-1 inhibitors expressed by activated immune cells. CD28 molecules expressed by T cells give out a costimulatory signal to APC B7 molecules, while CDLA-4 interrupts this costimulatory signal between activated T cells and APCs, thus suppressing the immune system. Therefore, CTLA-4 inhibitors reverse the immune suppression and T cell responses against tumors cells. PD-1 interacts with two ligands, such as PD-L1 and PD-L2. PD-1 interacts with stimulated cytotoxic CD8^+^ cells, rendering them unable to perform cytotoxicity, while PD-1 inhibitors activate CTLs to respond against the tumorigenic cells (132).

The chemokines CXCL1, CXCL2, CXCL3, CXCL4, CXCL5, CXCL6, CXCL7, and CXCL8 interact with their respective receptors. This interaction facilitates the chemotaxis of various inflammatory cells toward the site of inflammation. CXCR2 promotes the migration of neutrophils from bone marrow to the circulation and recruits the neutrophils to the site of inflammation. Myeloid-derived suppressor cells (MDSCs) are also influenced by CXCR2. CXCR2 is implicated not only in cancer development but also facilitates the process of metastasis. CXCR2 is essential for colon and skin tumorigenesis and lung cancer metastasis. CXCL1 and CXCL2 produced at the site of cancer enhance MDSC infiltration and metastasis (133). The role of the triad of neutrophils, MDSCs, and CXCR2 has been implicated in pancreatic tumors, and an elevated neutrophil count showed poor outcome in patients with pancreatic tumors (133). Furthermore, an increased level of CXCL5 chemokines not only promotes metastasis but is also related to worse prognosis (133), while CXCR2 inhibitors regress pancreatic tumor development in mice model (133).

IL-24 is synthesized by various cells including monocytes, T cells, B cells, dermal keratinocytes, and by stimulated colonic sub-epithelial myofibroblasts under the influence of IL-1 cytokines (134). IL-24 induces cancer cell death at high concentrations, triggers autoimmune responses, cardiovascular protection, inhibits keratinocyte growth, and possesses antibacterial activity (134, 135). IL-24-binding receptors such as Sigma-1 receptor (Sig1R) are highly expressed by cancerous cells and are involved in inflammation and cancer-related apoptosis. In view of the above discussion, Sig1R could potentially be a target for both inflammatory disease and cancer treatments. Recent evidence suggests that Sig1R is highly expressed on tumor cells and is associated with cancer invasion and metastasis. Correspondingly, IL-24 interaction with Sig1R offers a possible novel approach to cancer therapy *via* targeting IL-24 Sig1R. IL-24 exerts an antitumor effect by sensitizing transformed cell chemotherapy, facilitating cancer cells apoptosis, and hindering the process of angiogenesis (134). IL-24 inhibits angiogenesis by decreasing the count of the CD31^+^ angiogenic marker on T cells. In addition, IL-24 suppresses the synthesis of angiogenic factors including FGF, VEGF, TGF-β, and IL-8 synthesized by cancer cells, in turn inhibiting the differentiation of endothelial cells (136). Similarly, IL-24 also inhibits metastasis by inhibiting the synthesis of focal adhesion protein-2 and -9 and amplifying the synthesis of E-cadherin membrane family receptors. E-cadherin receptors are responsible for cell-to-cell adhesion (134). IL-2 is fundamental for the differentiation of naïve T cells into either effector or memory cells, and for Treg cell maintenance and maturation. IL-2 also regulates NK cells and T cell development and maturity. IL-2-mediated stimulation of NK cells and cytotoxic T cells secrete IFN-γ, which further facilitates the infiltration and activation of T cells into the tumor microenvironment. IFN-γ not only favors cancer cell apoptosis but also increases MHC-1 and MHC-2 molecule expression, thus helping the cytotoxic cells to recognize the tumor cells expressing the CD80 marker and target for cytotoxicity (115). Furthermore, the administration of IL-2 can lead to durable, complete, and apparently curative regressions in patients with metastatic melanoma and renal cancer (137).

VEGFRs are conjugated with tyrosine kinase activity intracellularly. Drugs such as sunitinib that target VEGFRs have improved overall survival in patients with RCC (138). IL-6 and TNF*-*α are often synthesized together during inflammation, stress, RA, and are even induced together by IFNs. Antibodies directed against TNF*-*α are considered as a first target and have proved clinically effective against RA (139, 140). Elevated levels of IL-6 have been observed in pathological conditions including RA, juvenile idiopathic arthritis (JIA), IBD, allergic asthma, multiple sclerosis, and serum lupus erythematosus (SLE). Monoclonal antibody blockade of IL-6 proved effective in the treatment of RA and JIA. However, anti-IL-6 monoclonal antibodies are under trial for the treatment of SLE and Crohn’s disease (139, 140).

## Vaccine-Based Immunostimulatory Approaches toward Cancer

Over time, the concept of a link between cancer and immune system has emerged. This concept lead to the use of bacillus Calmette–Guérin (BCG) in mice with an implanted tumor, which confers protection against tumor progression (141–143). Consequently, BCG injection under the direction of endoscopy into the bladder lesion caused successful complete remission (CR) of melanoma secondary to bladder tumor metastasis. BCG use as an immunotherapeutic agent was first reported by Alvaro Morals in 1976 and is the most successful event in the history of tumor immunotherapy, used against urothelial carcinoma (UC). It is still one of the most fascinating areas in ongoing urological research. BCG is considered a standard therapeutic modality in patients with non-muscle invasive bladder cancer with high risk and provides evidence that the immunotherapeutic approach in UC could be effective. Immunotherapy offers one of the best options for the treatment of bladder carcinoma, owing to several reasons, such as that bladder tumor is associated with a greater mutation rate and exhibits a high antigenic nature (141–143). Similarly, the immunotherapeutic agent is administered directly into the bladder (intravesical route), causing selective tumor cell death. Despite being used as immunotherapeutic agent in various malignancies, its exact mechanism is still unknown (141). However, it is proposed that BCG stimulates the immune system and triggers an inflammatory response (141–143). Following these events, BCG becomes attached and internalized *via* macropinocytosis by uroepithelial cells, and in response, increases MHC-2 molecule and cytokine expression. Under the influence of cytokines, the tumor microenvironment is infiltrated by lymphocytes. The lymphocytic infiltration is dominated by Th1, which is associated with the release of cytokines that induce the activation of CTLs and NK cells to exert cytotoxic activity. Several strains of BCG modified by recombinant technology have been develop that cause the synthesis of IL-2, IL-12, IL-18, IFN-α, and IFN-γ. With the intention to enhance its therapeutic potential, it is, however, currently in the preclinical phase and no human trials have yet been conducted (141–143).

Another immunomodulatory approach is the use of oncolytic viruses. Oncolytic viruses cause the death of the specific cells in which it is replicating and releases additional oncolytic viruses and tumor antigens. The serotype-5 oncolytic adenovirus CG0070 has been developed and tested in mice. These viruses specifically replicate in cells deficient for the RB pathway and have the gene for GM-CSF, an immunomodulatory cytokine. This virus vaccine, during a phase 1 trial in 35 human subjects with UC after intravesical administration, demonstrated that with a 10.4-month median duration, 49% of patients responded completely. In patients with RB status, the response was higher than 80%, whereas in those receiving six weekly doses, the response was 70%. However, phase 2/3 trials are underway for its complete evaluation (141, 142, 144). Correspondingly sipuleucel-T is the first FDA-licensed cellular immune therapy vaccine against non-viral cancer (145). The vaccine-based cellular approach composed of autologous or allogeneic tumor cells, which have limited immunogenicity and are genetically engineered to express cytokines, chemokines, and costimulatory signaling molecules. In particular are cytokines such as IL-4 and GM-CSF and costimulatory molecule CD80, which stimulate APCs to increase the expression of tumor antigens. As compared to immunization with GM-CSF, IFN-α and IL-12 peripheral immunization of rats with IL-4-transfected 9L cells achieved greater therapeutic outcome in rats having 9L gliosarcoma in preclinical studies. Similarly, IL-4 amplifies the Th1-type antitumor response when secreted locally at the vaccine site (125), and a more potent response was observed when IFN-α was locally administered along with IL-4 in intracranial tumor (125, 146).

Similarly, two viral vaccines are exploited for ovarian vaccine tumor treatment. One group of vaccines targets the “cancer-testis” antigen NY-ESO-1, engineered into fowlpox (rF) as a booster and vaccinia (rV) as a prime and as a vaccination. A phase 2 clinical trial of the stated vaccine in 22 subjects with advanced ovarian cancer expressing NY-ESO-1, associated with high risk of recurrence, showed encouraging results (147). The second group of viral genetic vaccines tested in ovarian cancer (PANVAC-C + PANVAC-V) involved a poxviral vaccine that is genetically engineered with CEA-MUC1-TRICOM (B7.1, ICAM-1, LFA-3) into fowlpox (PANVAC-C) as a booster and vaccinia (PANVAC-V) as a prime vaccination. The second genetic vaccine tested in ovarian cancer (PANVAC-C + PANVAC-V) was a poxviral vaccine, in which the CEA-MUC1-TRICOM (B7.1, ICAM-1, LFA-3) was engineered into vaccinia (PANVAC-V) as a prime and fowlpox (PANVAC-C) as a booster vaccination. Currently, these vaccines are also under clinical trials, in different phases (130).

## Targeted Approaches toward Cancer

Currently, available therapeutic agents have an inadequate distribution and pharmacokinetic profiles. The anticancer drugs used have a small molecular weight and are thus rapidly distributed and cleared from the circulation (148, 149). Owing to rapid clearance, anticancer drugs are eliminated before showing sufficient intended therapeutic action, while poor accumulation in cancerous tissue and distribution to non-cancerous tissues is associated with high toxicity (149). Thus, various strategies have been devised to make cancer therapy more effective by targeting specifically expressed biomarkers including protein, lipid, mutated genes, carbohydrate, and RNA and by using nanoparticulate systems (150). Nanoparticles concentrate in tumor tissue passively *via* an enhanced permeation and retention effect. This accumulation of drug (nanoparticle) in the tumor microenvironment is facilitated by the inappropriate angiogenesis related to the tumor (151). Angiogenesis is an important hallmark of cancer progression. Thus, the process of angiogenesis can be inhibited by using a liposome-based targeted drug delivery system (152). In addition, the discovery of cancer stem cells (CSCs) has been a major milestone in the development of effective therapies against cancers (153). Normal stem cells and CSCs share similar properties *via* differentiation into all cell types. However, CSCs are tumorigenic cells, and all types of cells are present only within the tumor microenvironment (153, 154). CSCs not only differentiate into multiple cell types but also maintain their population at similar levels to normal stem cells (153). Researchers have focused on CSCs owing to their ability to generate new tumor cells. Thus, CSCs are a major hurdle for tumor CR and are associated with relapses. The use of conventional cytotoxic agents to target CSCs is lacking specificity, and increasing the dose also increases the risk of adverse effects (153, 154). However, these problems could be overcome by using either nanoparticles or increasing the transport efficiency of drug into the tumor microenvironment. The transfer of drug into the cell is affected by the transporter that pumps the drug out of the cells and hence decreases its concentration within the cell. One of the most important drug transporters is the ABC transporter system, which pumps the diffused drug out of the cell (155, 156). However, nanoparticles enter the cell by the process of endocytosis and by bypassing these transport mechanisms. In addition, nanodrugs can be exploited to target cancer cells, either actively or passively (155, 156). Active drug delivery involves the binding of ligand directed against specific receptors, while passive targeting involves altering the blood supply toward the tumor microenvironment (155, 156).

The standard therapeutic protocol against the advanced small cell lung cancer is cispaltin + etoposide (C + E); however, most patients treated with this protocol have limited survival rate and showed higher relapse rate (157). However, this protocol proved fruitful when used in combination with the histone deacetylase inhibitor belinostat an epigenetic agent and exhibited less side effects (157). Furthermore, the tumor necrosis factor-related apoptosis-inducing ligand has been implicated in inducing the process of apoptosis and the recombinant TRAIL (rTRAIL) was evaluated by Kim et al. (158) against colitis-associated cancer (CAC) (158). The early rTRAIL administration was related to the significant inhibition of the colitis and CAC *via* reducing the infiltration of the macrophages into the mucosa of the colon, inducing the scavengering with efferocytosis and the synthesis of several growth factors compared with the late rTRAIL administration (158). Furthermore, the rTRAIL administration tends to accelerate the process of tissue regeneration and trigger the resolution of inflammation by NLRP3 (NACHT, LRR, and PYD domains-containing protein 3) inflammasome pathway. The study revealed that rTRAIL can be used as chemopreventive agent because of inhibition of the colitis and colitis-related cancer rather than using as therapeutic agent (158).

The resistance offered to the conventional chemotherapy is the major problem against the breast cancer. The microRNA-27b-3p (miR-27b) is a miRNA which is absent/deleted in the tissue of the patients with breast cancer and breast cancer cell lines which makes these cells chemoresistance, while its presence is related to the sensitization of the breast cancer cell to various chemotherapeutic agents (159). This miR-27b administration reversed the resistance to paclitaxel treatment and produced synergistic effect *via* direct interaction with the CBLB and GRB2 gene to inhibit the anti-apoptotic PI3K/Akt and MAPK/Erk signaling cascade and the combination of miR-27b with other chemotherapeutic agent have shown promising result against the breast cancer (159). The conventional anticancer therapies are based on the inducing the cell apoptosis. But most cancer cells show resistance to the anticancer therapy and are defective in apoptotic induction (160). Thus, to deal with this debilitating condition, the researchers have focused their attention on natural products such as Matrine, Saponins, Glabridin, and Platycodin D, which exhibit non-apoptotic programmed cell death (160). In addition, ribosome-inactivating proteins (RIPs) were exploited as potent cytotoxins that have potential anticancer properties. Mistletoe lectin 1 (ML1) is a heterodimeric cytotoxic protein present in *European Mistletoe* and belongs to RIP class II (161). This compound mediates it anticancer activity *via* uptake into (i) glycan binding on the cell surface; (ii) clathrin-dependent and -independent endocytosis; (iii) redirection of the protein from endocytic vesicles to Golgi network and presumably its subsequent retrograde transport to the ER (161). The ML1 follows this pathway for interacting with its molecular target to confer anti-proliferative and pro-apoptotic activity. Thus, this targeted approach can be used for eradication of multidrug resistant tumor cells which respond poorly to anticancer drugs that are transported via ABC transporters (including P-glycoprotein and multidrug resistance proteins) (161).

The endogenous immunity such as circulating anticancer antibodies or tumor reactive B cells within the cancer patients is historic and partly described concepts (162). Thus, by identifying the specific tumor antigens, the discovery of antitumor antibody can be a breakthrough in the field of cancer immunotherapy (162). Moreover, the efficacy of the most anticancer drugs is related to DNA damage induction. The cancer tissues and cells associated with the expression of abnormal type of genes and the product of these abnormal genes are vital for the DNA repair mechanism offer favorable target for the synthetic lethality (163). This occurs when there is inactivation of two genes concurrently or the product of these genes induces cell death; however, the inactivation of these genes individually is not lethal in nature. The evolution of this concept will lead to the developing of effective and personalized anticancer therapy (163). Currently, most of the tumors are treated by the systemic administration of anticancers. However, the efficacy of these chemotherapeutic is hampered by various attributes (164). To improve the drug delivery to the tumor microenvironment (cell to tissue scale or single cell pharmacology), microscale pharmacokinetics/pharmacodynamics (microPKPD) modeling framework was adopted. This microPKPD modeling approach takes into account the explicit tumor tissue morphology, its metabolic landscape and/or specific receptor distribution (164).

## Future Prospective

The appropriate role of the immune system in both inflammation and cancer is not yet absolutely established. However, immune cells inhibit or promote both inflammation and cancer depending on the type of cells involved and an inflammatory and tumor microenvironment. Furthermore, cytokines released by different cells in response to cancer and inflammation exert a plethora of effects on inflammation and the cancer prognosis. These cytokines also modulate responses of the immune cells involved in inflammation and tumor surveillance. Recently, several therapeutic approaches have been developed to target the immune cells and inflammatory cytokines. The basic aim of all these strategies is to augment the response of the immune system toward inflammation and cancer. Immunotherapeutic approaches using immune cells or targeting immune cells involved in cancer and inflammation offer novel approaches to treat these anomalies. Currently, cell- and vaccine-based therapies are under different stages of development; during initial trials, they have shown positive outcomes. NK cells, T cytotoxic cells, and T helper cells are the focus of these therapies. Similarly, Treg immunosuppressant cells implicated in pathogenesis of several tumors and inflammation also offer a novel approach to treat several tumors. Immune cells loaded with antitumor drugs are also exploited for the treatment of various malignancies. In addition, the inhibitory molecules expressed by immune cells such as PD-1 and CTLA-4 expressed on cytotoxic T cells have been a focus of researchers to treat various tumors. Consequently, vaccine-based therapies are also evaluated to combat an inflammatory and tumorigenic environment. BCG and several other vaccines are currently in clinical use, in one way or the other, for the treatment of tumors and other diseases. Currently, several monoclonal antibody therapies directed against cytokines are in clinical use, while many more are under different stages of development. Similarly, oncolytic viruses targeting tumors cells comprise another approach to deal with inflammation and cancer. However, to put the role of the immune system in inflammation and cancer into proper perspective requires further evaluation.

## Author Contributions

All the authors contributed equally to this work.

## Conflict of Interest Statement

The authors declare that the research was conducted in the absence of any commercial or financial relationships that could be construed as a potential conflict of interest.
